# Itaconate and Its Derivatives Repress Early Myogenesis *In Vitro* and *In Vivo*

**DOI:** 10.3389/fimmu.2022.748375

**Published:** 2022-02-21

**Authors:** Tae Seok Oh, Damian C. Hutchins, Rabina Mainali, Kevin H. Goslen, Matthew A. Quinn

**Affiliations:** ^1^ Department of Pathology, Section on Comparative Medicine, Winston-Salem, NC, United States; ^2^ Department of Internal Medicine, Section on Molecular Medicine Wake Forest School of Medicine, Winston-Salem, NC, United States

**Keywords:** C2C12, myogenesis, itaconate, succinate, succinate dehydrogenase

## Abstract

A Krebs cycle intermediate metabolite, itaconate, has gained attention as a potential antimicrobial and autoimmune disease treatment due to its anti-inflammatory effects. While itaconate and its derivatives pose an attractive therapeutic option for the treatment of inflammatory diseases, the effects outside the immune system still remain limited, particularly in the muscle. Therefore, we endeavored to determine if itaconate signaling impacts muscle differentiation. Utilizing the well-established C2C12 model of *in vitro* myogenesis, we evaluated the effects of itaconate and its derivatives on transcriptional and protein markers of muscle differentiation as well as mitochondrial function. We found itaconate and the derivatives dimethyl itaconate and 4-octyl itaconate disrupt differentiation media-induced myogenesis. A primary biological effect of itaconate is a succinate dehydrogenase (SDH) inhibitor. We find the SDH inhibitors dimethyl malonate and harzianopyridone phenocopie the anti-myogenic effects of itaconate. Furthermore, we find treatment with exogenous succinate results in blunted myogenesis. Together our data indicate itaconate and its derivatives interfere with *in vitro* myogenesis, potentially through inhibition of SDH and subsequent succinate accumulation. We also show 4-octyl itaconate suppresses injury-induced MYOG expression *in vivo*. More importantly, our findings suggest the therapeutic potential of itaconate, and its derivatives could be limited due to deleterious effects on myogenesis.

## Introduction

It is well appreciated that dramatic metabolic reprogramming occurs in response to inflammatory challenges. One of the most robust metabolic adaptations made in myeloid cells in response to inflammatory cues is the diversion of cis-aconitate away from α-ketoglutarate towards the synthesis of itaconate (1, 2). One primary function itaconate serves is to reduce inflammation *via* the inhibition of succinate dehydrogenase (SDH) thereby reducing accumulation of reactive oxygen species (ROS) (1). Due to its potent anti-inflammatory properties, itaconate and its derivatives such as dimethyl itaconate (DMI) and 4-octyl itaconate (4-OI) have gained attention as potential treatments for autoimmune conditions such as psoriasis (3), multiple sclerosis (4), rheumatoid arthritis (5), and systemic lupus erythematosus (6).

While the signaling attributes of itaconate with regards to immune function are well delineated, not much is known on the actions of itaconate in non-immune organs. What is known is that itaconate possesses anti-inflammatory, anti-fibrotic and pro-survival attributes in organs such as the liver (7) and kidney (8). Furthermore, amassing evidence has shown that itaconate derivatives have therapeutic effects on idiopathic pulmonary fibrosis (9), ischemia-reperfusion injury (10, 11), abdominal aortic aneurysm (12), and sepsis (13). However, the effects of itaconate regulation of muscle function are yet unknown.

Recent studies have shown side effects of commonly used drugs on muscle atrophy (14) and toxicity (15, 16). Nevertheless, knowledge concerning potential side effects of itaconate and its derivatives on muscle is still limited. Given that chronic inflammation is hypothesized to incur sustained itaconate production, coupled to its potential therapeutic use for the treatment of a variety of diseases, it is of the upmost importance to understand potential off target tissues and effects in the face of elevated itaconate levels. To address this critical gap in knowledge we utilized the well-established C2C12 murine myoblast model system for *in vitro* myogenesis as well as barium chloride-induced muscle regeneration and studied the effects of itaconate and its derivatives on altering myoblast differentiation.

## Results

### Itaconate Derivatives Inhibits Myogenesis in C2C12 Cells

**Figure 1:**
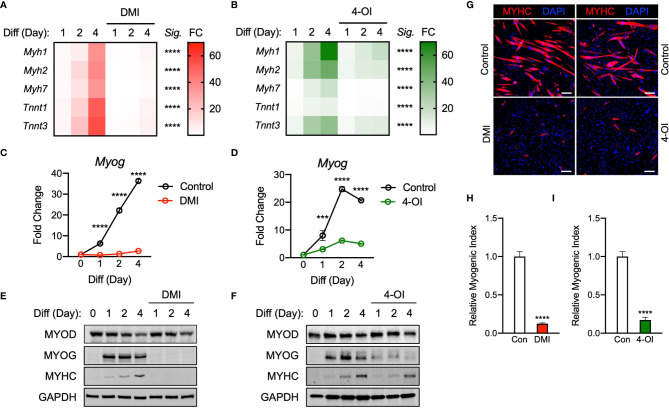
Itaconate derivatives repress C2C12 myogenesis by modulating myogenic transcription mechanisms. Heatmap depiction of average fold change (*Sig.* denotes statistical differences at day 4, n = 3) in myogenic gene transcription levels detected *via* RT-qPCR when exposed to differential media with or without **(A)** dimethyl itaconate (DMI: 125 µM) and **(B)** 4-octyl itaconate (4-OI: 125 µM) relative to the untreated group over a 4-day time course. Fold change of *Myog* mRNA expression at various time points comparing differentiation media (DM) control groups to **(C)** DMI and **(D)** 4-OI groups relative to expression without either treatment (n = 3). Western blotting data showing differences in myogenic transcription factor expression between DM treated groups and groups treated with DM plus **(E)** DMI and **(F)** 4-OI over a 4 day time course as well as groups which were not exposed to either treatment for any period. **(G)** MYHC staining at day 4 of differentiation. Bar: 100 μm. Relative myogenic index (percentage of total nuclei associated with myotubes) of **(H)** DMI and **(I)** 4-OI. *** denotes *p* < 0.001, ****p < 0.0001 compared to control based on t-test.

Past research has shown that Krebs cycle metabolites serve as signal transducers in the immune response within macrophages (17). We endeavored to evaluate how the presence of itaconate affects myogenic differentiation within C2C12 cells. Given its recent focus as a potential treatment for autoimmune conditions, the function of dimethyl itaconate (DMI) was first investigated. Gene expression markers for myogenesis (i.e. *Myh1*, *Myh2*, *Myh7*, *Tnnt1*, and *Tnnt3*) were measured in the presence of DMI versus vehicle over a 4-day differentiation protocol. We looked at day 1 and 2 to confirm initiation and induction of myogenesis and investigated day 4 to analyze myotube formation. As expected, vehicle group cells had a time-dependent increase in the transcription of myogenic genes (**Figure 1A**). However, treatment with DMI significantly inhibited the induction of these myogenic markers during our differentiation protocol (**Figure 1A**). Another analog of itaconate was also utilized since DMI cannot be endogenously converted to intracellular itaconate (18). 4-octyl itaconate (4-OI) was subsequently used because it has been suggested as a possible treatment for autoimmune conditions, but unlike DMI, it can be endogenously converted to itaconate (18, 19). We analyzed C2C12 cells treated with 4-OI over 4 days in the presence of differentiation media (DM). 4-OI significantly inhibited the induction of myogenic gene transcription as well (**Figure 1B**).

To further determine how DMI and 4-OI impairs myogenesis, we next assessed the expression of the essential myogenic transcription factors *Myog* and *Myod.* The expression of *Myog* mRNA was significantly impaired by DMI throughout day 1 to day 4 of differentiation compared to the control (**Figure 1C**). Transcription of *Myog* was also blunted in response to 4-OI exposure (**Figure 1D**). Protein levels of MYOD were not different in the DMI groups compared to the control in response to DM. However, MYOG expression was almost completely abolished by DMI (**Figure 1E**). Consequently, DMI-treated C2C12 myoblasts failed to differentiate into myotubes. DMI-treated groups showed no trace of MYHC in response to DM cues, while vehicle-treated cells displayed a time-dependent increase in MYHC protein (**Figure 1E**). 4-OI significantly blocked MYOG and MYHC expression levels although it did not change MYOD levels (**Figure 1F**). MYHC was visualized by immunofluorescence at day 4 of differentiation (**Figure 1G**) and myogenic index was significantly impaired by DMI (**Figure 1H**) and 4-OI (**Figure 1I**).

### Physiological Itaconate Inhibits Myogenesis

**Figure 2:**
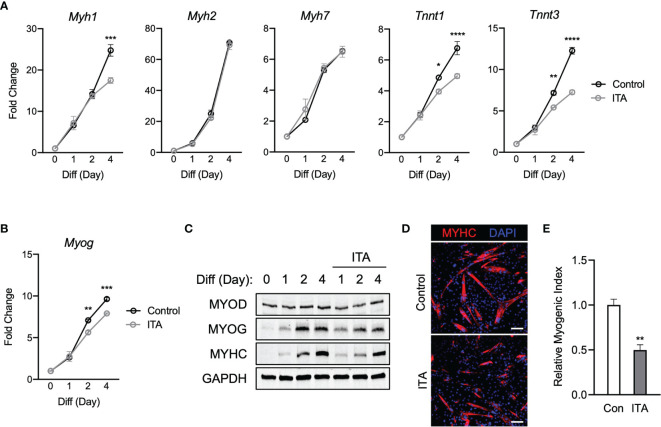
Itaconate represses C2C12 myogenesis by modulating myogenic transcription mechanisms. **(A)** 4-day time course showcasing fold change differences of myogenic gene transcription levels detected *via* RT-qPCR when exposed to differential media with or without itaconate (7.5 mM) relative to the untreated group (n = 3). **(B)** Fold change of *Myog* mRNA expression at various time points comparing DM control groups to DM and itaconate groups relative to expression without either treatment (n = 3). **(C)** Western blotting data showing differences in myogenic transcription factor expression between DM treated groups and groups treated with both DM and itaconate over a 4 day time course as well as groups which were not exposed to either treatment for any period. **(D)** MYHC staining at day 4 of differentiation. Bar: 100 μm. **(E)** Relative myogenic index (percentage of total nuclei associated with myotubes). * denotes *p* *<* 0.05, **p < 0.01, ***p < 0.001, ****p < 0.0001 compared to control based on t-test.

To investigate a role of physiological itaconate, C2C12 myoblast cells were then treated with DM only or DM plus itaconate and then examined over 4 days. It was reported that exogenous itaconate readily enters cells (17). Itaconate was used to portray physiological mechanisms more accurately. Transcription levels of *Myh2* and *Myh7* were not significantly impacted by itaconate exposure. However, the blunted induction of *Myh1*, *Tnnt1*, and *Tnnt3* was significant on the 4th day of exposure (**Figure 2A**). Significant inhibition of *Myog* transcription on days 2 and 4 was also observed in itaconate groups (**Figure 2B**). MYOG and MYHC expression on day 2 were also inhibited in itaconate groups (**Figure 2C**). Itaconate treatment reduced MYHC staining (**Figure 2D**) and myogenic index (**Figure 2E**).

Overall DMI shows the strongest myogenic inhibitory response and itaconate has the weakest amount of myogenic suppression among itaconate and derivatives tested. Taken together, these results suggest that itaconate and its derivatives are sufficient to inhibit myogenesis in C2C12 cells, modulating myogenic regulating factors at the transcription and protein levels.

### Itaconate Derivatives Lead to Inhibition of Succinate Dehydrogenase and Accumulation of Intracellular Succinate During Myogenesis

**Figure 3:**
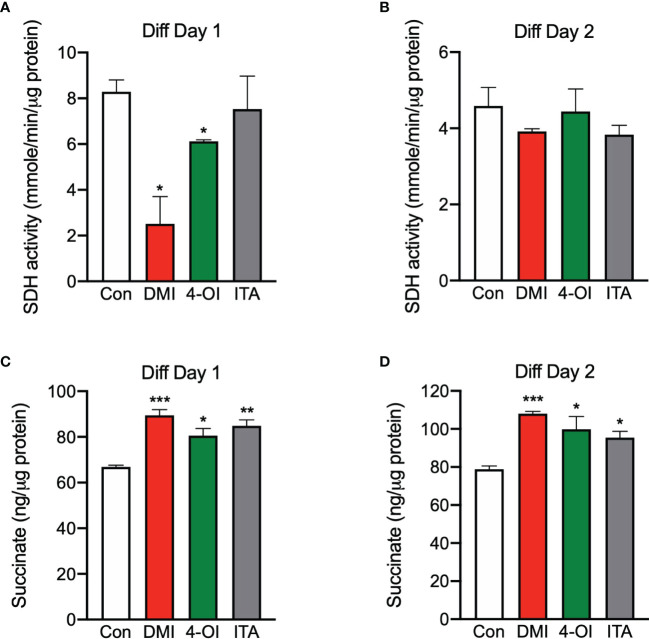
Itaconate derivatives inhibit succinate dehydrogenase and accumulate intracellular succinate during C2C12 myogenesis. Succinate dehydrogenase (SDH) activities in the presence of itaconate derivatives at day 1 **(A)** and day 2 **(B)** of differentiation (n = 6). Succinate levels in the presence of itaconate derivatives at day 1 **(C)** and day 2 **(D)** of differentiation (n = 6). * denotes *p* *<* 0.05, **p < 0.01, ***p < 0.001, ****p < 0.0001 compared to control based on t-test.

Considering itaconate can lead to accumulation of succinate *via* SDH inhibition (20–22), we tested if this happens in our model with itaconate derivatives. As expected, SDH activity was reduced significantly at day 1 of differentiation when treated with DMI and 4-OI compared to control (**Figure 3A**). However, this inhibition was not maintained at day 2 of differentiation (**Figure 3B**). In line with decreased SDH activity in response to itaconate derivatives during myogenesis, intracellular succinate levels were higher when treated with itaconate derivatives compared to control at day 1 and 2 of differentiation (**Figures 3C, D**). Taken together, inhibition of SDH by itaconate derivatives during myogenesis on day 1 was sufficient to keep succinate accumulated in C2C12 cells.

### Malonate and Pharmacological Succinate Dehydrogenase Inhibitor Obstruct Myogenesis

**Figure 4:**
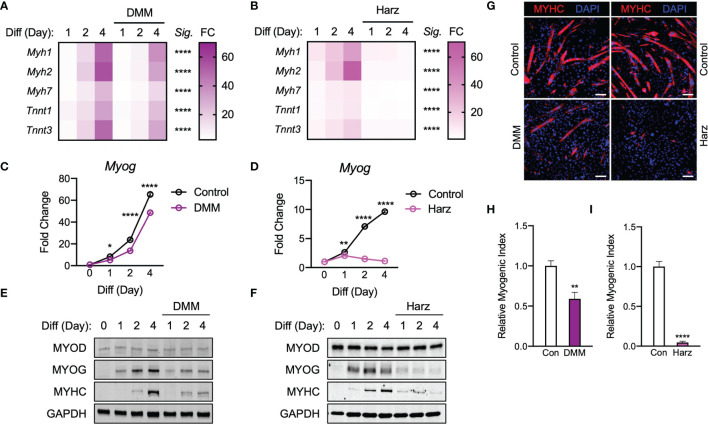
Malonate and pharmacological inhibitor of succinate dehydrogenase repress C2C12 myogenesis *via* similar mechanism of action to that of itaconate and its derivatives. Heatmap depiction of average fold change (*Sig.* denotes statistical differences at day 4, n = 3) in myogenic gene transcription levels detected *via* RT-qPCR when exposed to differential media with or without **(A)** dimethyl malonate (DMM: 5 mM) and **(B)** harzianopyridone (Harz: 4 µM) relative to the untreated group over a 4-day time course. Fold change of *Myog* mRNA expression at various time points comparing differentiation media (DM) control groups to **(C)** DMI and **(D)** 4-OI groups relative to expression without either treatment (n = 3). Western blotting data showing differences in myogenic transcription factor expression between DM treated groups and groups treated with DM plus **(E)** DMM and **(F)** Harz over a 4 day time course as well as groups which were not exposed to either treatment for any period. **(G)** MYHC staining at day 4 of differentiation. Bar: 100 μm. Relative myogenic index (percentage of total nuclei associated with myotubes) of **(H)** DMM and **(I)** Harz. * denotes *p* *<* 0.05, **p < 0.01, ****p < 0.0001 compared to control based on t-test.

Given that both itaconate and malonate are Krebs cycle metabolites that are shown to inhibit SDH (1, 23), our next step was to determine whether malonate exposure could also inhibit C2C12 myogenesis. Dimethyl malonate (DMM) was administrated instead of itaconate under similar parameters to those discussed in **Figures 1** through **2**. Malonate inhibited the induction of myogenic markers during myogenesis (**Figure 4A**). This inhibition was more severe than itaconate but not as robust as the inhibition caused by itaconate derivatives. In parallel, a potent and specific inhibitor of SDH, Harzianopyridone (Harz) (24), was tested to determine whether this specific shared inhibition was a mechanism underlying impaired myogenesis. We examined C2C12 cells exposed to Harz using the same methods to elucidate whether SDH inhibition independent of the Krebs cycle might also elicit a dramatic obstruction of myogenesis. Harz treatment resulted in significant suppression of myogenic markers (**Figure 4B**). Malonate lowered *Myog* levels compared to the control at all the time points (**Figure 4C**). Consistent suppression of *Myog* expression was also observed following Harz treatment throughout the time course (**Figure 4D**). MYOD levels were similar in malonate-treated cells during differentiation, but malonate led to a reduction in MYOG levels and a significant decrease in MYHC levels (**Figure 4E**). Harz showed significantly reduced MYOG and MYHC levels as well (**Figure 4F**). Malonate showed similar extent of reduction in MYHC staining (**Figure 4G**) and myogenic index (**Figure 4H**) as itaconate. Harz displayed the most robust inhibition of myogenesis shown by MYHC staining (**Figure 4G**) and myogenic index (**Figure 4I**). The degree of suppression caused by Harz was most comparable to the amount of inhibition by DMI. In tandem, these findings support that inhibition of SDH affects myogenesis in C2C12 cells.

### Myogenesis Is Inhibited by Exogenous Succinate

**Figure 5:**
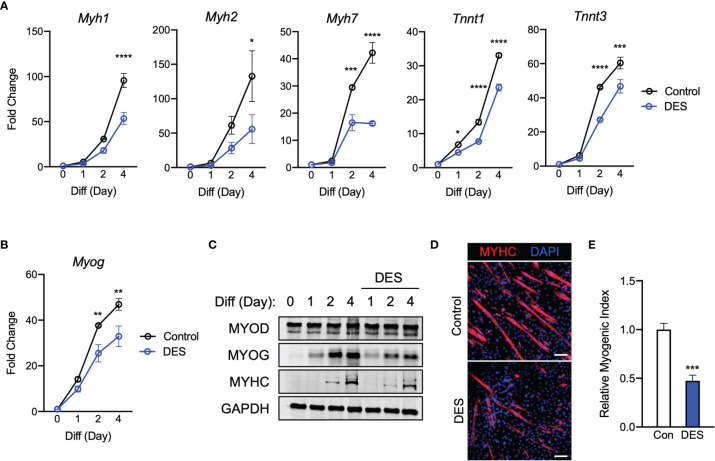
Elevated exposure to succinate elicits inhibition of C2C12 myogenesis *via* mechanism similar to those observed when exposing cells to itaconate and malonate. **(A)** 4-day time course showcasing fold change differences of myogenic gene transcription levels detected *via* RT-qPCR when exposed to differential media with or without diethyl succinate (DES: 5 mM) relative to the untreated group (n = 3). **(B)** Fold change of *Myog* expression at various time points comparing DM control groups to DM and DES groups relative to expression without either treatment (n = 3). **(C)** Western blotting data showing differences in myogenic transcription factor expression between DM treated groups and groups treated with both DM and DES over a 4 day time course as well as groups which were not exposed to either treatment for any period. **(D)** MYHC staining at day 4 of differentiation. Bar: 100 μm. **(E)** Relative myogenic index (percentage of total nuclei associated with myotubes). * denotes *p* *<* 0.05, **p < 0.01, ***p < 0.001, ****p < 0.0001 compared to control based on t-test.

Inhibition of SDH activity can lead to an increase in intracellular succinate levels (20). Given that SDH inhibition alone is able to significantly inhibit myogenesis, we next asked if succinate accumulation could be the connecting factor leading to myogenic inhibition. Thus, we tested if exogenous succinate could cause myogenic failure in C2C12 cells treated with DM. All the data with diethyl succinate (DES) represented a very similar trend to previous findings. Myogenic gene expression (**Figure 5A**), *Myog* (**Figure 5B**), and myogenic proteins (**Figure 5C**) were all inhibited by the exogenous succinate treatment. Reduced MYHC expression (**Figure 5D**) and myogenic index (**Figure 5E**) by succinate indicate its inhibitory potency of myogenesis. The degree of suppression was most similar to those results observed in groups treated with itaconate or malonate. These results support a possibility that inhibition of myogenesis by itaconate or malonate is at least partly due to accumulated succinate caused by SDH inhibition.

### 4-OI Inhibits Myogenic Induction During Post-Injury Muscle Regeneration

**Figure 6:**
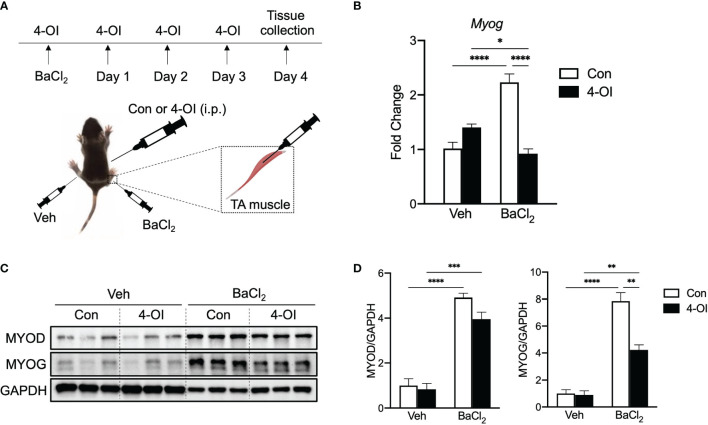
4-OI suppresses gene and protein expression of MYOG in muscle after injury. **(A)** Experimental scheme of muscle injury in mice. **(B)** Fold change of *Myog* mRNA expression at day 4 of muscle injury by barium chloride (BaCl_2_) with or without 4-OI (n = 4). **(C)** Western blotting data showing differences in myogenic transcription factor expression between vehicle (Veh) treated groups and BaCl_2_ treated groups with or without 4-OI. **(D)** Quantification of MYOD and MYOG. * denotes *p* *<* 0.05, **p < 0.01, ***p < 0.001, ****p < 0.0001 based on one-way ANOVA.

To gain insight on the effect of itaconate on myogenesis *in vivo*, we utilized a post-injury muscle regeneration model by injecting barium chloride (BaCl_2_) in TA muscle of mice and administrated 4-OI or control (**Figure 6A**). *Myog* gene expression was significantly increased on day four in injured TA muscle compared to vehicle (**Figure 6B**). Importantly, injury-induced *Myog* induction was blunted in the presence of 4-OI (**Figure 6B**). Muscle injury strongly increased protein levels of MYOD and 4-OI did not result in alteration of MYOD levels in BaCl_2_ treated muscle in accordance with *in vitro* findings (**Figures 6C, D**). In response to increases in MYOD, its downstream target MYOG was also elevated in injured TA muscle. Interestingly, protein levels of MYOG in 4-OI administrated mice were indeed significantly reduced compared to control in the BaCl_2_ group (**Figures 6C, D**). Collectively, these results indicate that 4-OI blunts myogenic induction during post-injury muscle regeneration *in vivo* by regulating one of the master myogenic transcription factors.

## Discussion

Itaconate has become the epitome of metabolites that also have immunomodulation properties. It is a decarboxylated cis-aconitate which is primarily known for its SDH inhibiting activity in the TCA cycle. While initially used as a polymer for industrial purposes, it has been researched to have both antimicrobial and anti-inflammatory properties. Some bacteria (e.g. *Yersinia pestis*) have itaconate degrading enzymes and itaconate itself has been observed to restrict the growth of bacteria by inhibiting isocitrate lyase activity (25). It also regulates host mechanisms. Its anti-inflammatory role has gained the most attention recently. Itaconate is observed to significantly increase in response to macrophage activation (23). It acts to reduce inflammation by inhibiting the release of proinflammatory cytokines through a plethora of mechanisms, inhibiting ROS production through suppression of SDH activity (1), activating the regulator of antioxidant expression NRF2 (26), and activating the anti-inflammatory signaling transcription factor ATF3 (3). These effects in tandem have attracted scientists to get approval for itaconate derivatives as potential treatments to limit microbial pathogenicity and to attenuate autoimmune disease symptoms.

DMI and 4-OI have been researched to have promising effects on psoriasis, rheumatoid arthritis, multiple sclerosis, and systemic lupus erythematosus due to their ability to suppress IL-17 signaling and reduce proinflammatory cytokine production (3, 6). While seemingly propitious as a potential treatment, the fact is that knowledge is very limited on the effects of itaconate and its derivates outside of those relating to the immunomodulatory axis. A consequence of itaconate derivative use may be one or more harmful side effects which could lessen enthusiasm for their utilization as treatments. Blocking itaconate signaling, which has yet to be proposed, might also be used to attenuate symptoms of other conditions. The focus of our research is to showcase the additional effects of itaconate on muscle regulation. Chronic inflammation has been linked to muscle wasting. Prolonged itaconate exposure resulting from chronic inflammation may cause or exacerbate muscle wasting.

Our hypothesis is that itaconate obstructs myogenesis *via* inhibition of SDH. This hypothesis arose from research done on the effects of succinate abundance on muscle wasting. Succinate is a substrate for SDH, so its intracellular concentration is directly linked to SDH activity. Succinate abundance has been proposed as a causative agent of muscle wasting. Treatments to elevate intracellular succinate concentration has been observed to decrease total muscle protein expression by ~25%, inhibit *in vitro* C2C12 myoblast cell myogenesis, decrease myofiber diameter in murine models, inhibit myogenesis *in vivo* following barium chloride injury, reduce respiration capacity by ~35% in myoblasts, and also disrupt a number of other metabolic processes within muscle cells (27). SDH has also been shown to have significantly reduced activity in sarcopenic muscle (28). Given that anti-inflammatory activity of itaconate is incurred *via* SDH inhibition, we endeavored to determine if itaconate treatment led to similar effects seen in succinate abundant cells.

We first tested the proposed treatment forms of itaconate, DMI, and 4-OI. We then examined itaconate alone followed by malonate, a pharmacological inhibitor of SDH, and then lastly diethyl succinate. These molecules did not show significant alterations in cell viability or proliferation, indicating inhibition of myogenesis by these molecules is independent of cytotoxicity (**Figure S1**). Treatment incurred transcriptional modulation of *Myh1, Myh2, Myh7, Tnnt1, and Tnnt3* along with alteration of myogenic transcription factor (mainly MYOG) and myogenesis marker MYHC protein expression were examined. Our results showcased substantial inhibition of myogenesis in C2C12 cells by DMI. 4-OI treatment also exhibited significant inhibition, but not as aggressive as DMI. Itaconate treatment also resulted in significant myogenesis inhibition, but not to the degree seen in either DMI or 4-OI treated groups. It is possible that inability of DMI conversion to itaconate leads to a strong inhibition of myogenesis exhibiting sustained activity because 4-OI can be converted to itaconate, but DMI cannot (18). It is established that itaconate can be converted to itaconyl-CoA by succinate-CoA ligase (21). A possible mechanism to explain this tiered order of inhibition severity may involve itaconate conversion to itaconyl-CoA. Regardless of potency, all itaconate treatments inhibited *in vitro* myogenesis.

We show that SDH suppression and subsequent succinate accumulation significantly inhibit myogenesis *via* myogenic transcription suppression. Given that MYOG expression is dependent upon MYOD activity, it is hypothesized that MYOD activity was also suppressed in the treatment groups, but *via* post translational modification that leads to changes in MYOD activity such as phosphorylation and acetylation (29, 30) rather than transcriptional inhibition. Inactive MYOD in response to SDH inhibition and succinate causes blunted induction of *Myog* thereby suppressing the transcription of myogenic genes. Together, our results support that itaconate may contribute to impairment of myogenesis *via* its effects on SDH which modulates myogenic transcription mechanisms. Therefore, future studies should be focused on elucidating further molecular mechanisms by which itaconate and its derivatives inhibit transcription of myogenic genes.

Sarcopenia patients have been observed to have inhibited mitochondrial functioning *via* transcriptional downregulation of key genes involved in oxidative phosphorylation and mitochondria proteostasis (28). 4-OI has been shown to increase aerobic respiration by reducing glycolytic function through inhibition of GAPDH (19). This data may implicate that in addition to subsequent muscle wasting induced by increased succinate concentration, prolonged itaconate activity may exacerbate issues relating to mitochondrial function. What demands further study is the determination of whether itaconate and its derivatives induce mitochondrial activity suppression which may cause subsequent inhibition of the oxidative phosphorylation pathway and thus reduce energy demand accommodation capability.

In this study, we find that itaconate and derivatives contribute to suppression of *in vitro* and *in vivo* myogenesis. Administrating 4-OI during post-injury muscle regeneration clearly contributes to suppression of MYOG in muscle of mice. It is speculated that skeletal muscle regeneration might be delayed in the presence of itaconate derivatives followed by lowered MYOG. Future studies about prolonged effects of itaconate during muscle regeneration will provide further insight about actual influence on muscle regeneration in the presence of itaconate. What remains to be researched next is how itaconate affects muscle regulation outside of succinate accumulation. For example, investigating the effects of succinylation on myogenic promotor binding proteins or transcription factors is warranted. Our results showcase functions of itaconate outside of immunomodulation and implicate that derivative use to treat autoimmune diseases or microbial infections should be cautioned due to a potential for deleterious effects on myogenesis. Further elucidation of these functions may lead to the development of treatments to attenuate muscle wasting *via* possible itaconate signaling inhibition.

## Materials and Methods

### Antibodies and Chemical Reagents

Primary antibodies used in this study are as follows: mouse monoclonal anti-GAPDH (Santa Cruz Biotechnology, sc-32233), mouse monoclonal anti-MYHC (Sigma, 05-716), mouse monoclonal anti-MYOD (Santa Cruz Biotechnology, sc-377460), and mouse monoclonal anti-MYOG (Santa Cruz Biotechnology, sc-12732). Reagents added to the media are as follows: Diethyl succinate (Sigma, 112402), Dimethyl itaconate (Sigma, 592498), Dimethyl malonate (Sigma 136441), Harzianopyridone (Santa Cruz Biotechnology, sc-280769), Itaconic acid (Sigma, I29204), and 4-octyl itaconate (Tocris Bioscience, 6662).

### Cell Culture and Differentiation

The mouse myogenic C2C12 myoblasts were maintained on plastic cell culture plates in Dulbecco’s modified Eagle’s medium (DMEM) supplemented with 10% fetal bovine serum and 1% penicillin-streptomycin in a humidified incubator kept at 37°C and 5% CO_2_. Cells were used up until passage 9. For differentiation, cells at 90% confluency were serum restricted with differentiation medium (DMEM, 2% horse serum, 1% penicillin-streptomycin) for up to 4 days. Growth or differentiation medium was replenished every day. For treatment in differentiation medium, following concentration was used and freshly added every day until the end of experiments: DMI (125 µM), 4-OI (125 µM), itaconate (7.5 mM), DMM (5 mM), Harz (4 µM), and DES (5 mM).

### RNA Isolation and RT-qPCR

RNA isolation was performed using the commercially available Aurum RNA miniprep kit (Bio-rad, 732-6820). Gene expression analysis was conducted with 50 ng RNA using iTaq Universal One-Step RT-qPCR Kit (Bio-rad, 1725140). The reaction was carried out according to the manufacturer’s instructions using CFX Connect Real-Time PCR Detection System (Bio-rad, 1855200). Probes used for TaqMan^®^ Gene Expression Assays (ThermoFisher) were as follows: *Myog* (Mm00446194_m1), *Myh1* (Mm01332489_m1), *Myh2* (Mm01332564_m1), *Myh7* (Mm00600555_m1), *Ppib* (Mm00478295_m1), *Tnnt1* (Mm00449089_m1), and *Tnnt3* (Mm01137842_g1).

### Western Blot

Cells were lysed in RIPA buffer (ThermoFisher, 89901) in the presence of Halt™ Protease and Phosphatase Inhibitor Cocktail (ThermoFisher, 78440), and protein concentration was measured using Pierce™ BCA Protein Assay Kit (ThermoFisher, 23227). Lysates were boiled for 5 min with 4x Laemmli Sample Buffer (Bio-Rad, 1610747) and 2-mercaptoethanol (Bio-Rad, 1610710). Lysates were resolved on SDS polyacrylamide gels and blotted onto PVDF membranes using Trans-Blot Turbo RTA Midi 0.45 µm LF PVDF Transfer Kit (Bio-Rad, 1704275). Transfer was run in Trans-Blot Turbo Transfer System (Bio-Rad, 1704150) using Mixed MW (1.3A-25V-7M) protocol. The membranes were blocked with Intercept™ (TBS) Blocking Buffer (LI-COR, 927-60001) for 1 h at room temperature and incubated with appropriate primary antibodies diluted 1:1000 in blocking buffer at 4°C overnight. After 3 washes with 0.05% tween20 (Bio-Rad) in TBS buffer (Bio-Rad), the membrane was incubated with IRDye^®^ 800CW Goat anti-Mouse IgG (LI-COR, 926-32210) and IRDye^®^ 680RD Goat anti-Rabbit IgG (LI-COR, 926-68071) secondary antibodies diluted 1:10000 in blocking buffer for 1 h at room temperature. Band images were visualized using ChemiDoc™ MP Imaging System (Bio-Rad, 12003154).

### Immunofluorescence and Calculation of Myogenic Index

Cells were seeded in a 24-well cell culture plate and followed by above-mentioned differentiation protocol. At day 4 of differentiation, cells were rinsed with PBS and fixed with 4% paraformaldehyde for 10 min at room temperature. Then, cells were permeablized with 0.5% Triton™ X-100 (Fisher, BP151-100) in PBS for 10 min at room temperature. Next, cells were submerged with Intercept^®^ (PBS) Blocking Buffer (LI-COR, 927-70001) for 1 h at room temperature. MYHC primary antibody was diluted to 1:100 in blocking buffer and incubated overnight at 4°C with the fixed cells. Next day, after 3 washes with 0.05% tween20 in PBS, Alexa Fluor 594 secondary antibody (Fisher, A11005) was diluted to 1:500 in blocking buffer and incubated with the cells for 1 h at room temperature. Followed by further washes, cells were mounted with VECTASHIELD^®^ Antifade Mounting Medium with DAPI (Vectalabs, H-1200) and covered with circular cover glass. Images were taken using ZOE™ Fluorescent Cell Imager (Bio-rad, 1450031). Myogenic index was calculated as the percentage of nuclei in fused myotubes (MYHC positive) out of the total nuclei in given images. Distribution of nuclei in myoblasts and myotubes was measured by counting over 100 nuclei at 4 distinct locations. The number of nuclei was counted using imageJ.

### Succinate Dehydrogenase Activity and Succinate Assay

SDH activity was measured using Succinate Dehydrogenase Assay Kit (Sigma, MAK197) according to manufacturer’s protocol. Briefly, at day 1 or 2 of differentiation in the presence of itaconate derivatives, cells were rinsed with PBS and lysed with SDH assay buffer. The whole cell lysate was freshly used for the measurement. SDH activity was calculated by measuring product absorbance at 600 nm every 5 min for 30 min at 25°C. SDH activity was normalized by protein contents in each sample measured by BCA assay. Succinate levels were determined using Succinate Colorimetric Assay Kit (Sigma, MAK184). After differentiation in the presence of itaconate derivatives, cells were rinsed with PBS and lysed with succinate assay buffer. The cell lysate was diluted 1:40 in assay buffer for analysis. After incubating samples with the reaction mix for 30 min at 37°C, the absorbance was measured at 450 nm. Concentration of succinate was normalized by protein contents in each sample measured by BCA assay.

### Muscle Injury in Mice

Male C57BL/6J mice aged 8 weeks were purchased from The Jackson Laboratory (Bar Harbor, ME). All animals were subjected to a 12:12 hr dark/light cycle with ad libitum access to standard rodent chow and water. To induce muscle injury *in vivo*, mice were anesthetized with 2-3% of isoflurane, and then 50 µl of 1.2% BaCl_2_ (Sigma, 202738) was slowly injected unilaterally into the TA muscle alongside tibia. Mice were kept warm during recovery and then returned to their cage. 4-OI (25mg/kg; Tocris, 6662) dissolved in saline plus (2-Hydroxypropyl)-β-cyclodextrin (Sigma, H107) was pretreated intraperitoneally 2 h beforehand and given daily for 3 consecutive days. At day 4, tissues were collected for further analysis. All protocols and experimental procedures were reviewed and approved by the Institutional Animal Care and Use Committee of Wake Forest School of Medicine (Winston-Salem, NC).

### Statistical Analysis

Unpaired t-test and one-way ANOVA using analysis software in Graphpad Prism 9 were performed to determine significant relationships between groups in the experiments. Data are represented as mean ± SEM.

## Data Availability Statement

The raw data supporting the conclusions of this article will be made available by the authors, without undue reservation.

## Author Contributions

TO planned, executed, analyzed data, and drafted manuscript. DH planned, executed, analyzed data, and drafted manuscript. RM executed, analyzed data, and drafted manuscript. KG executed and analyzed data. MQ planned, executed, analyzed data and drafted manuscript, and supervised the project. All authors contributed to the article and approved the submitted version.

## Conflict of Interest

The authors declare that the research was conducted in the absence of any commercial or financial relationships that could be construed as a potential conflict of interest.

## Publisher’s Note

All claims expressed in this article are solely those of the authors and do not necessarily represent those of their affiliated organizations, or those of the publisher, the editors and the reviewers. Any product that may be evaluated in this article, or claim that may be made by its manufacturer, is not guaranteed or endorsed by the publisher.
