# Supplementary figures and images for: Itaconate and Its Derivatives Repress Early Myogenesis In Vitro and In Vivo
Source: Front Immunol. 2022 Feb 21;13:748375. doi: 10.3389/fimmu.2022.748375 (PMC8898833; doi:10.3389/fimmu.2022.748375)

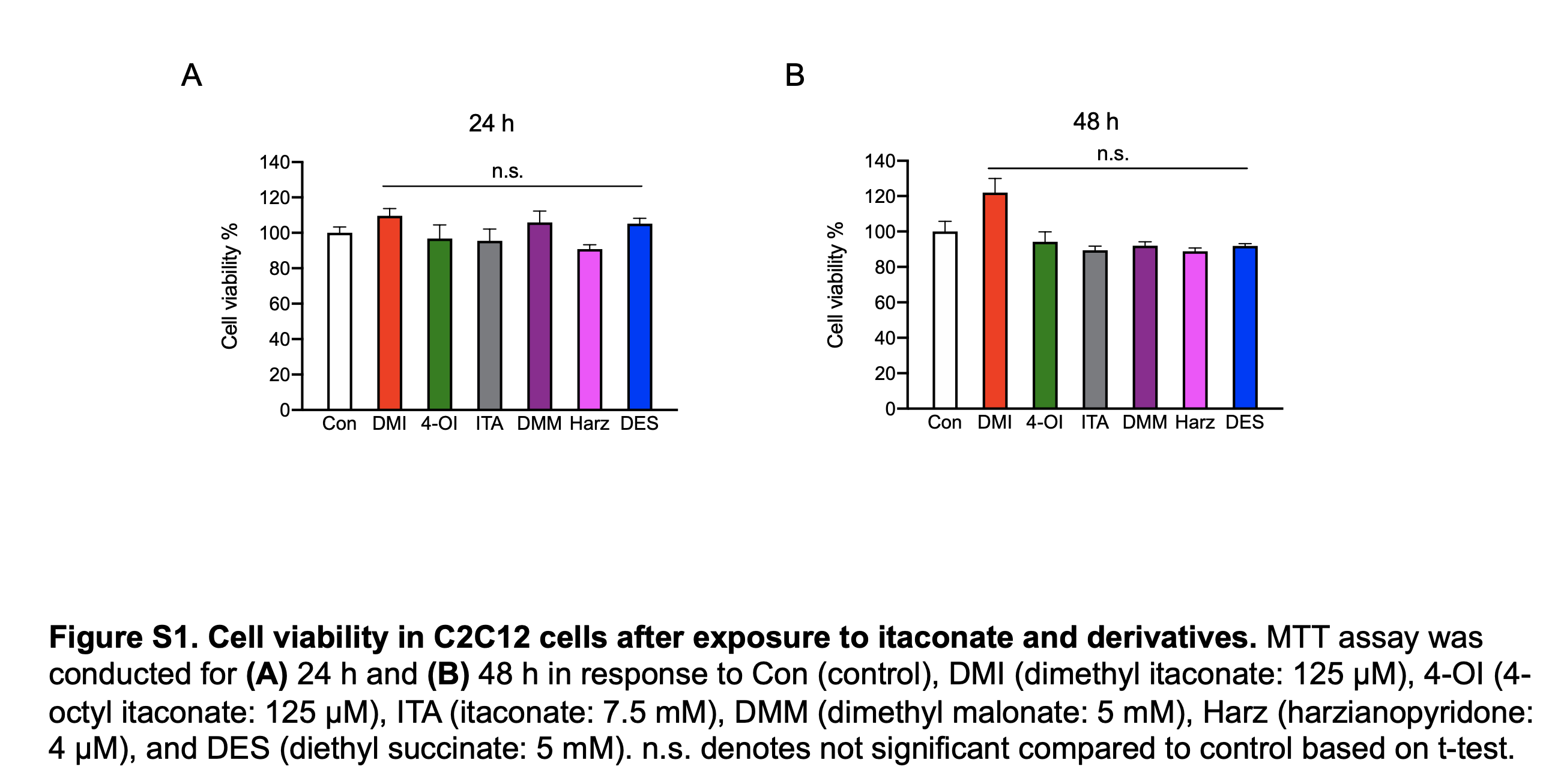

Supplement: Supplementary file 1 [file Image_1.tiff]
